# Iron Ion Particle Radiation Resistance of Dried Colonies of *Cryomyces antarcticus* Embedded in Martian Regolith Analogues

**DOI:** 10.3390/life10120306

**Published:** 2020-11-24

**Authors:** Lorenzo Aureli, Claudia Pacelli, Alessia Cassaro, Akira Fujimori, Ralf Moeller, Silvano Onofri

**Affiliations:** 1 Department of Ecological and Biological Sciences, University of Tuscia, Largo dell’Università snc, 01100 Viterbo, Italy; lorenzo.aureli@unitus.it (L.A.); cassaro@unitus.it (A.C.); onofri@unitus.it (S.O.); 2 Italian Space Agency, Via del Politecnico snc, 00133 Rome, Italy; 3 Molecular and Cellular Radiation Biology Group, Department of Basic Medical Sciences for Radiation Damages, National Institutes for Quantum and Radiological Science and Technology (QST), Chiba 263-8555, Japan; fujimori.akira@qst.go.jp; 4 German Aerospace Center, Institute of Aerospace Medicine, Radiation Biology Department, Aerospace Microbiology, DLR, Linder Höhe, D-51147 Köln, Germany; ralf.moeller@dlr.de; 5 Natural Sciences Department, University of Applied Sciences Bonn-Rhein-Sieg (BRSU), von-Liebig-Straße 20, D-53359 Rheinbach, Germany

**Keywords:** cosmic rays, accelerated iron ions, Mars, fungi, life on Mars

## Abstract

Among the celestial bodies in the Solar System, Mars currently represents the main target for the search for life beyond Earth. However, its surface is constantly exposed to high doses of cosmic rays (CRs) that may pose a threat to any biological system. For this reason, investigations into the limits of resistance of life to space relevant radiation is fundamental to speculate on the chance of finding extraterrestrial organisms on Mars. In the present work, as part of the STARLIFE project, the responses of dried colonies of the black fungus *Cryomyces antarcticus* Culture Collection of Fungi from Extreme Environments (CCFEE) 515 to the exposure to accelerated iron (LET: 200 keV/μm) ions, which mimic part of CRs spectrum, were investigated. Samples were exposed to the iron ions up to 1000 Gy in the presence of Martian regolith analogues. Our results showed an extraordinary resistance of the fungus in terms of survival, recovery of metabolic activity and DNA integrity. These experiments give new insights into the survival probability of possible terrestrial-like life forms on the present or past Martian surface and shallow subsurface environments.

## 1. Introduction

The search for life beyond Earth requires us to define the limits of resistance within which life can persist. In this context, one of the main limiting factors for any terrestrial form of life outside the terrestrial magnetic field is represented by the cosmic rays (CRs) [1]. CRs, which pervade the interstellar and interplanetary space, consist of totally ionized atomic nuclei accelerated at nearly the speed of light inside and outside our Solar System [2]. These highly energetic ions can penetrate any biological system, causing densely packed ionization events in the molecules along their path by the transferring energy to the crossed medium. For this reason, exposure to galactic cosmic rays (GCRs) can result in dramatic consequences in cellular structures and functions [3]. The average energy transferred from an accelerated ion to the penetrated medium per unit path length is described by the linear energy transfer (LET), and it increases with the square of the ionic charge [4]. Because CRs are totally ionized, high (H) atomic number (Z) energy (E) particles (HZE) represent the most damaging component of the CRs spectra, although they constitute only 1% of these [5].

The surface of celestial bodies lacking in a dense atmosphere and a global magnetic field are exposed to high doses of CRs. Among them, Mars is currently the main target for the search for extraterrestrial life due to the biocompatible environmental conditions during its early history and the accessibility for explorative missions [6]. Although there is no location on Earth reproducing exactly the Martian environmental conditions, some places such as the ice-free area of the McMurdo Dry Valleys in Antarctica have a combination of physicochemical parameters similar to those described on Mars [7]. Here, a few microorganisms have evolved strategies to withstand the extreme environmental factors; one of these is the cryptoendolithism, that is, the colonization of the small interstices inside porous rocks, where microorganisms can find protection from the external harsh conditions [8,9]. It is reasonable to speculate that, if Martian forms of life exist or ever existed, they may have adopted a similar strategy to cope with the extreme environment found on the planet [10]. In this regard, sandstones and subsurface environments might provide a shield against CR incident on the Martian surface.

In the frame of the study of the limits of resistance of the terrestrial life to CRs, an international consortium called STARLIFE was established in 2012, with the aim to test the biological responses of several selected extremophilic organisms to irradiations with high energetic ions representative of the CR spectrum [11]. Among the microorganisms tested in STARLIFE experiments, the Antarctic cryptoendolithic black fungus *Cryomyces antarcticus*, which lives inside the rocks in the McMurdo Dry Valleys, showed a high tolerance to accelerated He ions (150 MeV/n, LET 2.2 keV/μm) up to 1 kGy and γ-radiation (^60^Co) up to 55.61 kGy in desiccated conditions [12,13]. In distinct experiments, the fungus was also able to resist to acute irradiation with deuterons up to 1.5 kGy and X-rays up to 0.3 kGy, in metabolically active conditions [14] and to protracted X-ray exposure [15]. Additionally, the microorganism survived 18 months real space exposure and Martian simulated conditions, and therefore space radiation environment, in the form of LIFE (lichens and fungi experiment) and BIOMEX (BIOlogy and Mars EXperiment) experiments [16,17,18].

Here, the responses of dried colonies of *C. antarcticus* to the exposure to HZE particles iron (Z = 26) up to 1 kGy during the second STARLIFE-irradiation campaign, were investigated. Although iron ions only constitute 17.8 × 10^−3^% of the totality of the CRs [5], their high LET make them some of the most damaging particles in the CR spectrum.

The aim of the work is to investigate the effects of the presence of Martian dry regolith analogues on the survival of the fungus *C. antarcticus* after irradiation with accelerated iron ions. The regoliths are phyllosilicatic mars regolith simulant (P-MRS), which mimics the regolith of phyllosilicate deposits mainly observed on early Mars, and sulfatic mars regolith simulant (S-MRS), which is an analogue of regolith present in Martian sulphate deposits. The fungal responses were assessed on different levels by (i) a cultivation test (CFUs count); (ii) metabolic activity assessment (MTT assay); (iii) membrane damage assessment (PMA assay); (iv) DNA damage assessment (single gene PCRs, quantitative qPCR and fingerprinting analysis).

## 2. Materials and Methods

### 2.1. Samples Preparation and Exposure Conditions

The Antarctic cryptoendolithic strain of the black fungus *C. antarcticus* Culture Collection of Fungi from Extreme Environments (CCFEE) 515 was isolated from Antarctic sandstone [9]. Fungal colonies were grown (2000 colony-forming units, CFUs) on malt extract agar (MEA) medium in Petri dishes and incubated at 15 °C for 3 months. The colonies in the Petri dishes were dried under laminar flow in a sterile cabinet for one night, and subsequently detached from the medium for the exposure. Dry colonies were mixed to different materials and put in tubes with a volume of 200 μL (10 colonies per tube). Four sets of samples were thus obtained (Figure S1):

(i) Only dried colonies, with no materials (here referred as directly exposed cells).
(ii) Dried colonies mixed with grinded Antarctic sandstone, that is the original substratum (OS) where the fungus naturally occurs.
(iii) Dried colonies mixed with phyllosilicatic mars regolith simulant (P-MRS).
(iv) Dried colonies mixed with sulfatic mars regolith simulant (S-MRS).

The mineralogical composition of the Martian analogues is reported in Table S1. All materials were previously dried-sterilized (140 °C for 4 h). Depending on the position of each colony in the tubes, OS, P-MRS and S-MRS exhibited a shielding thickness ranging from 0 to 1.48 g/cm^2^, 1.55 g/cm^2^ and 1.53 g/cm^2^, respectively. This configuration allowed to simulate the Martian surface and shallow subsurface environment.

Colonies were separately irradiated with different doses of accelerated iron (Fe) ion particle irradiation (with an energy of 500 MeV/n (nucleon), LET in water: 200 keV/μm) using the heavy ion medical accelerator (HIMAC) at the National Institutes for Quantum and Radiological Science and Technology (QST) in Chiba, Japan. The applied doses were 50, 250, 500 and 1000 Gy and the dose rate of 12 Gy/min. Controls (non-irradiated samples) were treated in an identical manner as the irradiated samples. All tests were performed in triplicate.

### 2.2. Survival Assessment

#### 2.2.1. Cultivation Test

Microorganisms survival after ions exposure was assessed through the count of developed colonies related to the number of CFUs spread on MEA medium in Petri dishes. Three colonies from each sample were rehydrated at 15 °C in 1 mL of physiological solution (NaCl 0.9%) for 72 h; 0.1 mL of suspensions (50,000 cells/mL) were spread on Petri dishes supplemented with MEA in quintuplicate. Samples were incubated at 15 °C for 3 months and formed colonies were counted. Means and standard deviations were calculated for every replicate series. Statistical analyses were performed by one-way analysis of variance (Anova) and pair wise multiple comparison procedure [19], carried out using the statistical software SigmaStat 2.0 (Jandel). The Pearson correlation factors between the dose exposure and the growth colonies were calculated.

#### 2.2.2. Metabolic Activity Assessment by MTT Assay

In order to evaluate cells metabolic activity, the MTT (3-(4,5-dimethylthiazol-2-yl)-2,5-diphenyltetrazolium bromide) assay was performed. Cell suspensions were put in quadruplicate into 96-well microplates and 100 μL of 0.5 mg/mL MTT salt in phosphate-buffered saline (PBS) were added to each well. Four wells were filled only with MTT solution. After incubation in the dark at room temperature for 48, MTT solution was removed and 100 μL of dimethyl sulfoxide was added to each cell suspension. The absorbance was read at 595 nm, and the average absorbance of wells containing only MTT was subtracted from the others. Mean and standard deviations were determined for each quadruplicate and results were normalized with the laboratory controls.

#### 2.2.3. Membrane Damage Assessment

Quantitative PCR (qPCR) in DNA from samples treated with propidium monoazide (PMA) was performed to assess the membrane integrity in exposed samples. An aliquot of each rehydrated sample was added with 5 μL of a PMA solution (200 μM) and incubated in the dark for 1 h with occasional shaking. Samples were then placed in ice and exposed to a halogen lamp for 10 min. PMA can selectively penetrate cells with damaged membranes and cross-link to DNA under exposure to light, thereby preventing the polymerase chain reaction (PCR). DNA extraction and purification were performed on PMA treated and untreated aliquots from each sample. DNA was quantified and normalized at same concentration (2 ng/mL) using Qubit dsDNA HS Assay Kit (Life Technologies, Carlsbad, CA, USA) and qPCR was performed to quantify the number of fungal internal transcribed spacer (ITS) ribosomal DNA fragments (281 bp) present in both PMA-treated and untreated samples, according to the work of [16]. All tests were performed in triplicate.

### 2.3. DNA Integrity Assessment

#### 2.3.1. DNA Extraction, Single Gene PCR Reactions and RAPD Analysis

DNA extraction was performed on dried colonies using NucleoSpin^®^ Plant kit (Macherey-Nagel, Düren, Germany) following the protocol optimized for fungi [20]. Quantitation of extracted genomic DNA was performed using Qubit system and all the samples were diluted to the same concentration (2 ng/mL). Three overlapping tracts in the internal transcribed spacer (ITS) regions and the large subunit-coding sequences (LSU) of the nuclear ribosomal RNA (rRNA) gene complex were amplified. The used primers were ITS4a (ATTTGAGCTGTTGCCGCTTCA), ITS5 (GGAAGTAAAAGTCGTAACAAGG), LR5 (TCCTGAGGGAAACTTC) and LR7 (TACTACCACCAAGATCT). PCR reactions were carried out for each sample in a solution consisting of 12.5 μL of BioMixTM (BioLine Ltd., London, UK), 1 μL of each primer solution (5 pmol/μL) and 0.2 ng of DNA template, in a final volume of 25 μL. MyCycler Thermal Cycler (Bio-Rad Laboratories GmbH, Munich, Germany) equipped with a heated lid was used and amplification conditions are as reported in [21]. The whole genome integrity was assessed by random amplified polymorphic DNA (RAPD). PCR reactions were carried out for each sample in a final solution containing 12.5 μL of BioMixTM, 5 pmol of primer (GGA)_7_ and 0.2 ng of DNA sample, in a final volume of 25 μL. Amplifications were performed according to [21].

#### 2.3.2. Quantitative Assay of DNA Damage by qPCR

Quantitative PCR was carried out to quantify the number of ITS fragments by using LR0R (ACCCGCTGAACTTAAGC) and LR5 primers. The qPCR reactions were performed in triplicate with a solution containing 7.5 μL of qPCR cocktail (iQ SYBER Green Supermix, Biorad, MI, Italy), 1 μL of each primer solution (5 pmol/μL) and 0.1 ng of DNA template in a final volume of 15 μL. The amplifications were carried out by Biorad CFX96 real time PCR detection system.

#### 2.3.3. DNA Mutation Detection

Sequencing of a tract with a length of 1440 bp localized in rRNA gene region was performed to assay the presence of small DNA mutation after irradiation. Sequencing was performed by Macrogen Inc. (Spanish branch, Avda. Sur del Aeropuerto, 28, Madrid, Spain) using ITS4 and ITS5 primers. Alignment among obtained sequences from all the samples was carried out by using Molecular Evolutionary Genetics Analysis version 7.0 (MEGA 7). Sequences were also hierarchically clustered using the Unweighted Pair Group Method with Arithmatic Mean (UPGMA )method by MEGA 7.

## 3. Results

### 3.1. Survival Assessment

#### 3.1.1. Cultivation Test

**Figure 1:**
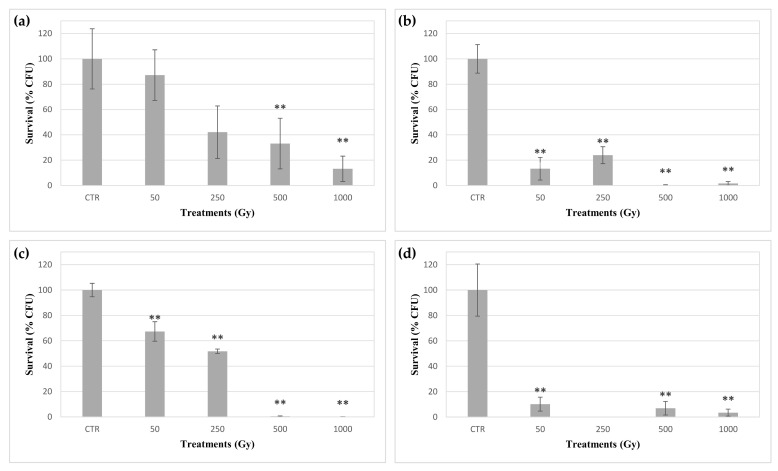
Survival of *C. antarcticus* exposed to accelerated iron ions expressed as CFUs. (**a**) directly exposed colonies, and colonies mixed with (**b**) original substratum (OS), (**c**) phyllosilicatic mars regolith simulant (P-MRS) analogue, (**d**) sulfatic mars regolith simulant (S-MRS) analogue. CTR: control. Data were normalized against the control. Significant differences were calculated by Tukey test with * = *p* < 0.05 and ** = *p* < 0.001.

The ability of *C. antarcticus* cells to grow after the exposure to accelerated iron ions was investigated by counting the number of colonies formed in the MEA medium. As shown in Figure 1, samples exposed to ions in absence of any material exhibited a progressive dose-dependent decrease in growth, with a Pearson correlation coefficient value (r) of −0.90. Significantly, 13% of colonies were able to grow after exposure to a dose of 1000 Gy (Figure 1a). Differently, samples exposed to iron ions in the presence of the three distinct materials (i.e., OS, P-MRS, S-MRS) showed different trends with a significant drop in growth colonies, compared to the respective controls, starting from the dose of 50 Gy (Figure 1b–d). Moreover, a correlation between the colony number and the exposure dose was reported for samples exposed while mixed to P-MRS analogue (r = −0.88). Notably, the growth ability was maintained at the highest dose of 1 kGy in every set, with a percentage of developed colonies of 1.6, 0.13 and 3.5% in the presence of OS, P-MRS and S-MRS, respectively (Figure 1b–d).

#### 3.1.2. Metabolic Activity Assessment by MTT Assay

**Figure 2:**
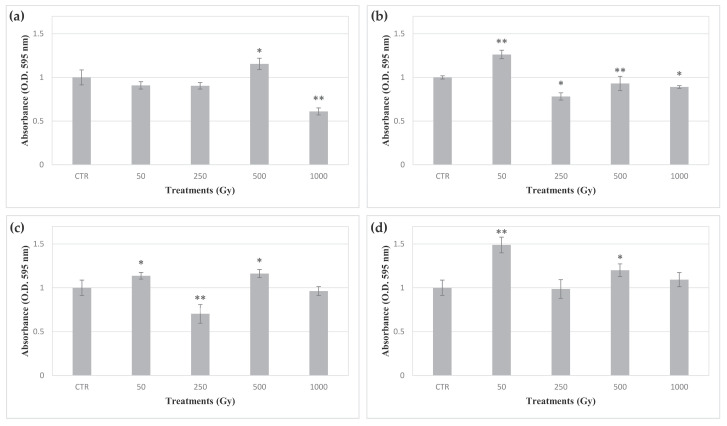
Effect of accelerated iron ions irradiation on metabolic activity recovery of *C. antarcticus*. (**a**) directly exposed colonies, and colonies mixed with (**b**) OS, (**c**) P-MRS analogue, (**d**) S-MRS analogue. CTR: control. Statistical analyses as reported in Figure 1. * = *p* < 0.05 and ** = *p* < 0.001.

MTT dye, a yellow water-soluble compound, is reduced to purple colored formazan crystals by mitochondrial succinate dehydrogenase in metabolically active cells. The DMSO dissolved formazan can be quantified spectrophotometrically at a wavelength of 595 nm, with the absorbance being linearly correlated with the metabolic activity of the cells in the sample. Here, MTT assay was performed to assess the metabolic activity recovery of *C. antarcticus* cells exposed to accelerated iron ions, after 48 and 72 h of rehydration. All samples showed a detectable metabolic activity, and samples from each set exhibited slight differences according to the irradiation dose (Figure 2). In samples rehydrated for 48 h after exposure in OS, P-MRS and S-MRS, a common trend with an increase in formazan absorbance corresponding to a dose of 50 Gy, followed by a decrease at the dose of 250 Gy and a new increase at the dose of 500 Gy was observed. 

#### 3.1.3. Membrane Damage Assessment

**Figure 3:**
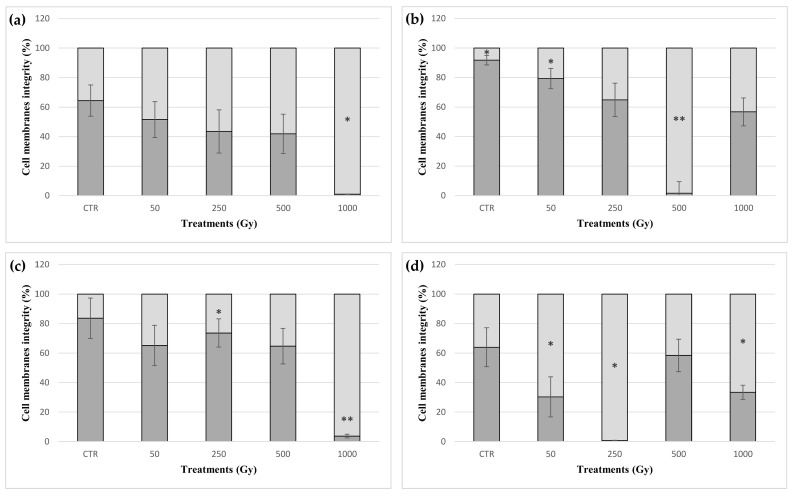
Percentage of intact and damaged cell membranes measured with propidium monoazide (PMA) assay coupled with qPCR of *C. antarcticus* exposed to accelerated iron ions. (**a**) directly exposed colonies, and colonies mixed with (**b**) OS, (**c**) P-MRS analogue, (**d**) S-MRS analogue. Light grey bars represent the percentage of cells with damaged cell membranes; dark grey bars represent the percentage of cells with un-damaged cell membranes. CTR: control. Statistical analyses as reported in Figure 1. * = *p* < 0.05 and ** = *p* < 0.001.

The PMA assay combined with qPCR was used to discriminate compromised membrane to membrane-intact cells. PMA is a compound that can covalently bind to DNA only in cells with a compromised cell membrane, thereby preventing PCR amplification. Figure 3 shows the ratio between cells with damaged membranes and cells with intact membranes after the exposure to increasing doses of accelerated iron ions. No significative damaged membranes were detected at the lowest doses in directly exposed cells, even though a sharp drop of cells with intact membrane- at 1 kGy was observed (1% of all cells) (Figure 3a). A similar drop at 1 kGy dose was only observed in cells exposed in the presence of P-MRS (4% of intact-membrane cells) (Figure 3c). Membrane damage in cells exposed in the presence of OS, P-MRS and S-MRS did not show any apparent correlation with the dose, although a reduction in cells with intact membranes was observed in most of the samples exposed to iron ions. In OS and S-MRS samples, a steep increase in damaged cell membranes at the dose of 500 and 250 Gy was observed (98.4 and 99.3% cells with damaged cell membranes, respectively) (Figure 3b,d).

### 3.2. DNA Integrity Assessment

**Figure 4:**
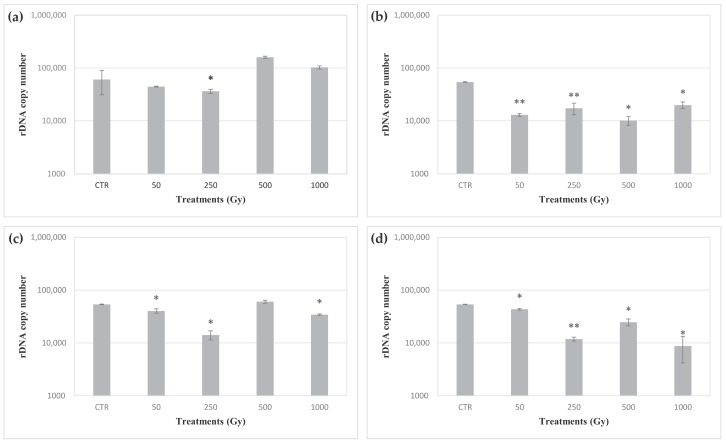
rDNA (internal transcribed spacer (ITS)- subunit-coding sequences (LSU) region) copy number quantification by qPCR of DNA of *C. antarcticus* colonies exposed to accelerated iron ions. (**a**) Directly exposed colonies, and colonies mixed with (**b**) OS substratum, (**c**) P-MRS analogue, (**d**) S-MRS analogue. CTR: control. Statistical analyses as reported in Figure 1. * = *p* < 0.05 and ** = *p* < 0.001.

After exposure to accelerated iron ions, structural damage to DNA in *C. antarcticus* cells was assessed in three overlapping tracts in the ITS-LSU region with a length of 700, 1600 and 2000 bp. DNA amplification was observed both in controls and samples exposed to doses up to 1 kGy of iron ions in each set (Figures S2–S4). The whole genome integrity of the exposed cells was investigated by RAPD assay. No evident change in amplicon profiles was shown by cells exposed up to 1 kGy of iron ions in each sample’s set (Figure S5). To assess levels of eventual DNA damage in cells of *C. antarcticus*, a quantitative PCR assay was performed in a target sequence of 939 bp in length mapping in the LSU region. Figure 4 shows the number of amplicons detected in cells on *C. antarcticus* after the exposure to increasing doses of iron ions, performed directly and in the presence of OS substratum, P-MRS and S-MRS analogues. Significative differences were detected among the controls and the cells exposed to doses from 50 Gy to 1 kGy of accelerated iron ions in the presence of OS, P-MRS and S-MRS (Figure 4b–d). Instead, only a 500 Gy dose caused a significative reduction in the number of the amplicons in cells exposed to accelerated iron ions in absence of any material. In order to detect the alteration in DNA sequences after accelerated iron ions exposure, the sequencing of a target tract of 1440 bp in the ITS-LSU region was performed. The results showed that *C. antarcticus* DNA was not affected by any point mutation, maintaining the nucleotide sequence after all the exposure doses.

## 4. Discussion

Among the celestial bodies in our solar system, Mars represents the main target for the search for extraterrestrial life. However, due to the lack of a global magnetic field and a dense atmosphere [22,23], the surface of the planet is constantly hit by CRs, high energized ions which pervade the interplanetary medium. The continuous exposure to these energized particles experienced on the surface on Mars may represent one of the most harmful factors for any terrestrial-like form of life. Significantly, different types of CRs diverge in their biological effectiveness according to their energy deposition in the traversed medium, which is defined by the LET value [24]. In this regard, a positive correlation exists between the charge of the accelerated ions and the LET. Consequently, despite their low fluxes, HZE particles represent the most damaging component of the CRs spectrum. Additionally, high LET ions can travel shorter distances than lower LET ions in the penetrated matter.

Therefore, although the Martian surface is exposed to most parts of the CRs spectrum, HZE particles can be shielded by the top centimeters of the regolith [25], making the subsurface Martian environment suitable for colonization by putative extraterrestrial microorganisms. Conversely, any hypothetical microorganisms present on the surface of Mars or in the top few centimeters of the Martian regolith would be directly exposed to high doses of these extremely harmful particles. For this reason, it can be assumed that any hypothetical Martian form of life may have adopted an endolithic strategy or colonized the subsurface environment to survive the extremely harsh conditions reported on the surface of the planet [10]. However, due to the liquid water availability in transient brines in the uppermost centimeters of regolith [26] and the movement of the soil by wind [27], there may be a chance to find microorganisms on the surface of Mars. For this reason, understanding if the life can survive and persist in this environment may be of primary importance in the ambit of the search for life on Mars.

In this context, the present study aimed to test the effects of increasing doses (up to 1 kGy) of one of the most abundant CR HZEs, namely accelerated iron ions (Fe^26+^) [28], on dried colonies of the extremophilic black fungus *C. antarcticus*. The exposure was performed both directly and while mixed with small amounts of Antarctic sandstones and two Martian regolith analogues in order to reproduce part of the surface and the shallow subsurface environments of Mars. Dried cells were employed to simulate a scenario where hypothetical Martian microorganisms may persist in a metabolically dormant state for extended periods until the transient availability of liquid water. In this scenario, the microorganisms may accumulate high doses of HZE cosmic rays until the reactivation of the cellular metabolism. Therefore, from the point of view of the reactivated cells, the total amount of absorbed dose, and not the dose rate, is the critical factor.

In this experiment, rehydrated cells of *C. antarcticus* showed the ability to grow after the exposure up to 1 kGy of accelerated iron ions in the absence of any materials and while mixed to OS substratum and P-MRS and S-MRS analogues (Figure 1). Nevertheless, a clear correlation between the growth ability and the exposure dose was only shown by colonies exposed without materials and in P-MRS. Additionally, cells exposed in absence of materials exhibited a higher growth ability after exposition to higher doses than cells mixed with OS, P-MRS and S-MRS. This latter result was similar to those found in a previous study, in which dried colonies of *C. antarcticus* were exposed to accelerated helium ions [29]. The maximum shielding depths of grinded OS, P-MRS and S-MRS were 1.48 g/cm^2^, 1.55 g/cm^2^ and 1.53 g/cm^2^, respectively. Therefore, according to the LET of the accelerated iron ions (200 keV/μm in water), the more harmful effects of the exposure in the presence of the materials cannot be explained by the localization of the cells inside the Bragg peak region along the particle’s track. Instead, these results may be explained by the effects of the secondary particles produced by the collisions of the accelerated ions with the atoms in the materials. These secondary particles, such as pions and kaons, decay into neutrinos, muons, gamma rays and beta particles (electrons and positrons) [30,31]. The propagation of this radiation in the minerals surrounding the cells may both amplify the damaging effects of accelerated ions and affect the cells in a dose-unrelated fashion. In this context, the differences in the colonies growth among the different sets may be due to the distinct properties of OS, P-MRS and S-MRS materials. Alternatively, the production of reactive oxygen species (ROS) from the water absorbing minerals in the OS and the regolith simulants may have differently affected the cells in the distinct sets [32]. Compared to our previous results on the growth of *C. antarcticus* cells exposed to accelerated helium ions [29], these results showed that the fungus exhibited a lower resistance when directly exposed to the more damaging iron ions (Figure 1), in accordance with the different LET values among helium and iron ions. However, differently from the survival reported after accelerated helium ions in the presence of OS, P-MRS and S-MRS materials, no linear survival trend was reported in this experiment. These results are related to the interaction between the accelerated iron particles and the materials in which *C. antarcticus* colonies were exposed; these interactions (and the related secondary effects) are lower in helium ions.

The metabolic activity of *C. antarcticus* exposed cells was assessed after 48 h of rehydration by MTT assay. The MTT assay is a colorimetric test based on the detection of the enzymatic reduction of MTT to MTT-formazan by mitochondrial succinate dehydrogenase. Therefore, two main parameters may influence formazan production; the relative number of viable cells in the sample and mitochondrial respiration. In this respect, one of the main cellular responses after radiation exposure is represented by mitochondrial biogenesis, which leads to an increase in enzymatic MTT reduction [33]. An apparently irregular trend was observed in formazan absorbance among the samples in each set. In this case, the absence of a clear trend of dose-dependent alterations of metabolic activity may be explained through the simultaneous and opposite effects of the killed cell ratio and activation of response to irradiation by survived cells in each sample. Therefore, the higher metabolic activity of the damaged cells may result in an increase in the total amount of formazan in each sample, thus compensating for the reduced number of viable cells after exposure. In this regard, although cells exposed at 250 Gy of accelerated iron ions in the presence of S-MRS analogue did not exhibit growth, they showed metabolic activity. Such discordance may be explained by the inability of the survived cells to grow after three months incubation, due to the high levels of cellular damage.

All the DNA sequences mapped in the target ITS-LSU region showed amplification in cells exposed up to the highest doses (Figures S2–S4). The good maintenance of the DNA structural integrity at the target region was confirmed by qPCR assay (Figure 4). Similarly, a whole genomic analysis of exposed cells showed no significant changes in the RAPD profiles, up to a dose of 1 kGy, in any set of samples (Figure S5). Similarly, no point mutations were detected in the target sequence (1440 bp) mapping in the ITS-LSU region in any of the samples. Overall, these results show that the main cause of the cell mortality may not be only due to the production of single and double strand breaks in the DNA molecules, but also to damage to other cellular components. Instead, HZE exposure can led to the production of ROS and reactive nitrogen species (RNS) inside the cells [34]. In turn, these reactive species can promote the alteration of cellular structures, such as the cell membrane [35]. In this regard, the PMA assay showed that the relative number of membrane-damaged cells increased in most of exposed samples, albeit not in a dose-related manner, in accordance with the survival trend.

All the reported results show how dried colonies of the cryptoendolithic black fungus *C. antarcticus* can survive the exposure of a dose of up to 1 kGy of HZE particles, albeit exhibiting a significant reduction in colony growth at the highest doses. Through the LET value of the accelerated iron ions (200 keV/μm), it is possible to calculate the number of the particles that hit every sample at each exposure dose [36]. However, depending on the solar modulation and differences in atmospheric depth, CRs fluxes on the Martian surface can vary over time [37,38]. Therefore, an accurate estimation of the time frame required to absorb similar numbers of particles is not possible. Besides, HZE particles reaching the Martian surface have a wide range of energy values, whereas in the present experiment only particles with an energy of 500 MeV/n were employed. Nevertheless, our results give a rough estimate of the resistance of terrestrial life to HZE exposure in a Martian-like scenario. In this context, the volumes of regolith mixed with colonies of *C. antarcticus* used in our experiment would require approximately more than 10 million years to accumulate a dose of 1 kGy of accelerated iron ions on the Martian surface (Table S2). Therefore, the ability of dried colonies of *C. antarcticus* to reactivate their metabolism and growth after such dose, while maintaining the structural integrity of DNA, shows how a hypothetical *C. antarcticus*-like microorganism may tolerate the surface and shallow subsurface Martian environment for years. However, the differences occurring among the distinct sets of samples show how the responses to CR exposure may strongly depend on the material surrounding the cells. Because of the reduced depth of the material used in the exposed samples, these differences are not attributable to their shield from the accelerated ions. Instead, the distinct responses of the cells to CR exposure may be due to other processes, such as the production of secondary radiation and ROS, that may amplify—in an irregular manner—the effects of this kind of radiation on the cells.

## Supplementary Materials

The following are available online at https://www.mdpi.com/2075-1729/10/12/306/s1, Figure S1: Dried colonies of *C. antarcticus* mixed with distinct materials, Figure S2: PCR amplification of the internal transcriber spacer (ITS) region (600 bp), of samples accelerated iron ions from each set, Figure S3: PCR amplification of the ITS-large subunit region (LSU) region (1600 bp), of samples exposed to accelerated iron ions from each set, Figure S4: PCR amplification of the ITS-large subunit region (LSU) region (2000 bp), of samples exposed to accelerated iron ions from each set, Figure S5: random amplification of polymorphic DNA (RAPD) assay in *C. antarcticus* colonies after exposure to accelerated iron ions from each set, Table S1: phillosilicate mars regolith simulant (P-MRS) and sulfatic mars regolith simulant (S-MRS) analogues composition, Table S2: estimated resistance of dried colonies of *C. antarcticus* to HZE on the Martian surface and shallow subsurface.
