# Supplementary material for: Iron Ion Particle Radiation Resistance of Dried Colonies of Cryomyces antarcticus Embedded in Martian Regolith Analogues
Source: Life (Basel). 2020 Nov 24;10(12):306. doi: 10.3390/life10120306 (PMC7761078; doi:10.3390/life10120306)
Supplement: Supplementary file 1 [file life-10-00306-s001.pdf]

life

MDPI

Supplementary Material of Iron Ion Particle Radiation Resistance of Dried Colonies of *Cryomyces antarcticus* Embedded in Martian Regolith Analogues

Table S1. Phillosilicate Mars Regolith Simulant (P-MRS) and Sulfatic Mars Regolith Simulant (S-MRS) analogues composition (modified from [1]).

P-MRS		S-MRS	
**Mineral**	**Weight (%)**	**Mineral**	**Weight (%)**
Montmorillonite	45	Gabbro	32
Chamosite	20	Gypsum	30
Quartz	10	Dunite	15
Iron(III)-oxide	5	Hematite	13
Kaolinite	5	Goethite	7
Siderite	5	Quartz	3
Hydromagnesite	5		
Gabbro	3		
Dunite	2		

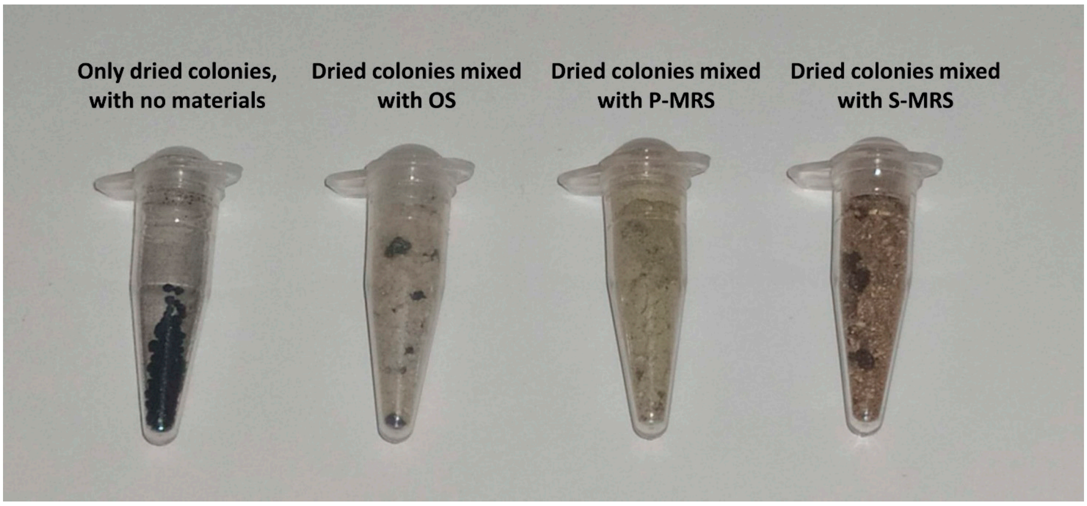

Figure S1. Dried colonies of C. antarcticus mixed with distinct materials. From the left: only dried colonies of C. antarcticus; dried colonies mixed with grinded Antarctic sandstone, that is the Original Substratum (OS); dried colonies mixed with Phyllosilicatic Mars Regolith Simulant (P-MRS), an analogue of phyllosilicate deposits mainly occurred on early Mars; dried colonies mixed with Sulfatic Mars Regolith Simulant (S-MRS), an analogue of regolith mainly observed in the current Martian sulphate deposits.

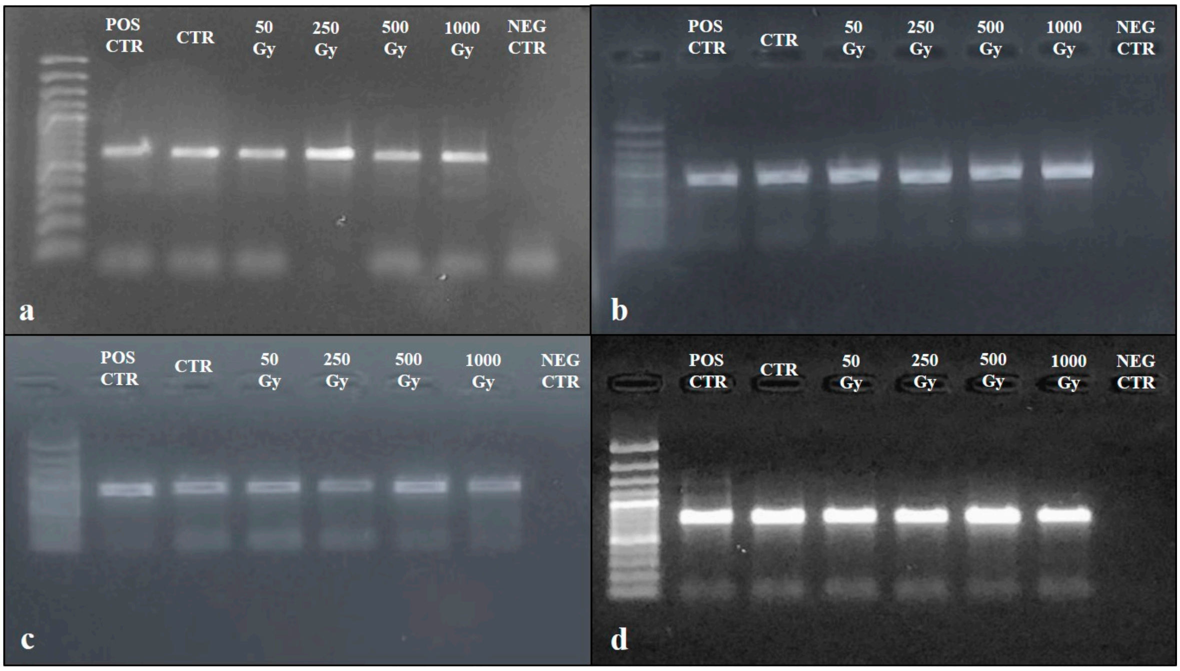

Figure S2. PCR amplification of the Internal Transcriber Spacer (ITS) region (600 bp), of samples accelerated Fe ions from each set: (**a**) directly exposed *C. antarcticus* colonies; (**b**) *C. antarcticus* colonies exposed in the (Original Substrate (OS) substratum; (**c**) *C. antarcticus* colonies exposed in the Phillosilicate Mars Regolith Simulant (P-MRS) analogue; (**d**) *C. antarcticus* exposed in the Sulfatic Mars Regolith Simulant (S-MRS) analogue. POS CTR: laboratory control; CTR: Control; NEG CTR: Negative Control.

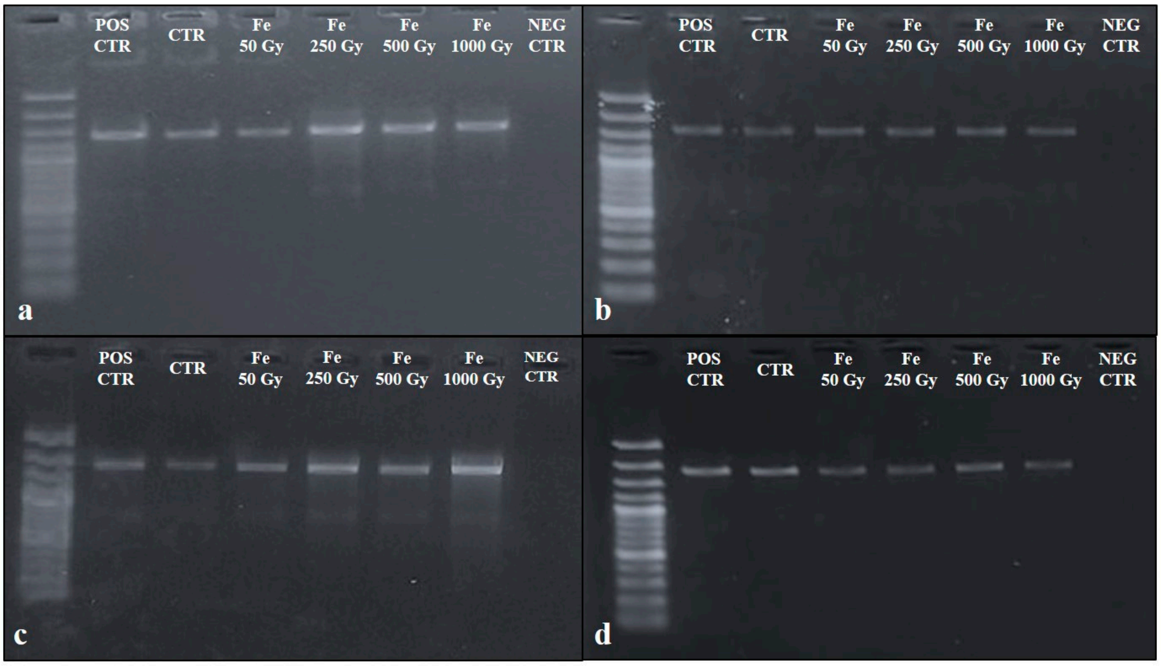

Figure S3. PCR amplification of the ITS- Large SubUnit region (LSU) region (1600 bp), of samples exposed to accelerated Fe ions from each set: (**a**) directly exposed *C. antarcticus* colonies; (**b**) *C. antarcticus* colonies exposed in the (Original Substrate (OS) substratum; (**c**) *C. antarcticus* colonies exposed in the Phillosilicate Mars Regolith Simulant (P-MRS) analogue; (**d**) *C. antarcticus* exposed in the Sulfatic Mars Regolith Simulant (S-MRS) analogue. POS CTR: laboratory control; CTR: Control; NEG CTR: Negative Control.

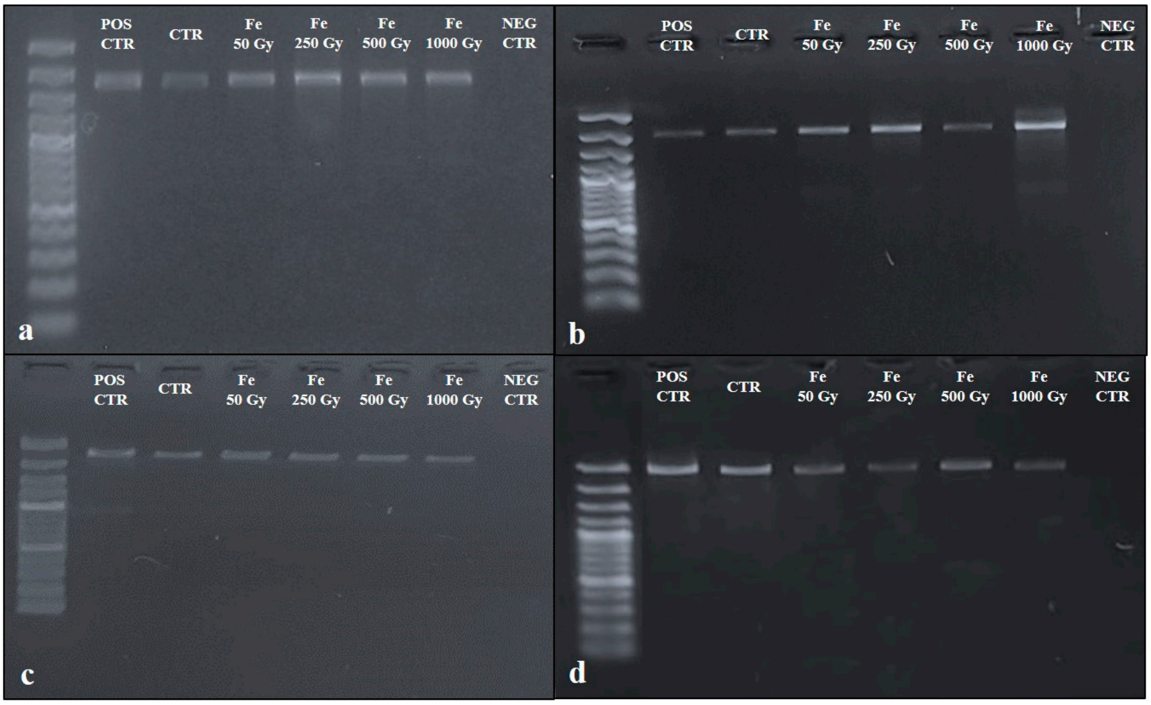

Figure S4. PCR amplification of the ITS- Large SubUnit region (LSU) region (2000 bp), of samples exposed to accelerated Fe ions from each set: (**a**) directly exposed *C. antarcticus* colonies; (**b**) *C. antarcticus* colonies exposed in the (Original Substrate (OS) substratum; (**c**) *C. antarcticus* colonies exposed in the Phillosilicate Mars Regolith Simulant (P-MRS) analogue; (**d**) *C. antarcticus* exposed in the Sulfatic Mars Regolith Simulant (S-MRS) analogue. POS CTR: laboratory control; CTR: Control; NEG CTR: Negative Control.

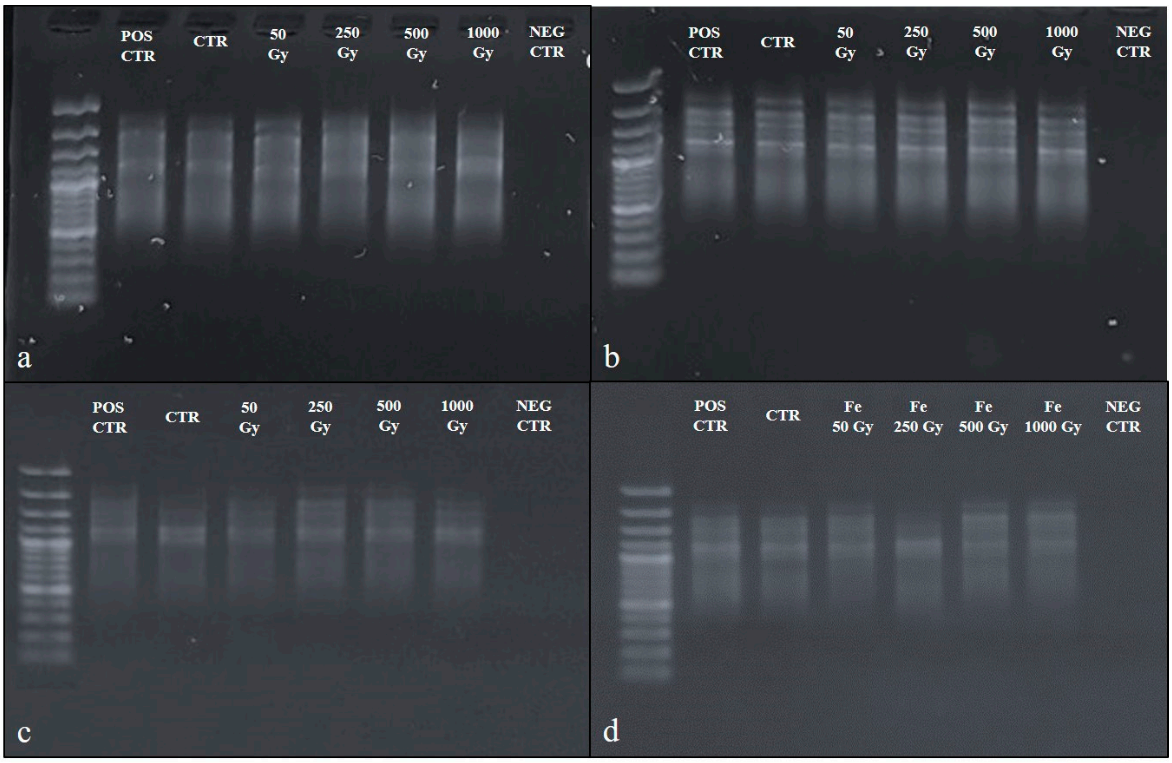

Figure S5. Random Amplification of Polymorphic DNA (RAPD) assay in *C. antarcticus* colonies after exposure to accelerated Fe ions from each set: (**a**) directly exposed *C. antarcticus* colonies; (**b**) *C. antarcticus* colonies exposed in the (Original Substrate (OS) substratum; (**c**) *C. antarcticus* colonies exposed in the Phillosilicate Mars Regolith Simulant (P-MRS) analogue; (**d**) *C. antarcticus* exposed in

the Sulfatic Mars Regolith Simulant (S-MRS) analogue. POS CTR: laboratory control; CTR: Control; NEG CTR: Negative Control.

Table S2 Estimated resistance of dried colonies of C. antarcticus to HZE on the Martian surface and shallow subsurface. Based on the survival of C. antarcticus colonies after accelerated Fe ions exposure, it is possible to determine the resistance of the fungus to HZE cosmic rays in a hypothetical Martian scenario. The second column shows the estimated number of the accelerated Fe ions that hit each sample (Martian regolith mixed to C. antarcticus colonies, 200 ml) in our experiment, calculated according to Aspen et al., 1994. In the third column, an estimation of the years required by the same volume of Martian regolith to absorb the calculated number of HZE ($Z \geq 26$) on Mars. Due to their relative abundances and high LET, accelerated Fe ions can be considered as representative of HZE cosmic rays reaching the Martian surface. The values were estimated by using the fluxes measured by The RAD Curiosity for CRs having atomic numbers ≥ 26, with the atmospheric depth of 21 g/cm^2 and the solar modulation parameter (F) of 577 MV [2]. The fourth column shows survival of C. antarcticus colonies, relative to the respective controls, at every dose of exposure in the distinct materials.

Table S2. Estimated resistance of dried colonies of *C. antarcticus* to HZE on the Martian surface and shallow subsurface.

Dose (Gy)	Fe Ions Fluence (Ions/Sample)	Time Exposure on Mars (Earth Years)	*C. antarcticus* Survival (% Colonies)
50	111×10^6	532×10^3	Directly exposed: 87.2; OS: 13.3; P-MRS: 67.3; S-MRS: 10.2
250	552×10^6	2.662×10^3	Directly exposed: 42.1; OS: 23.9; P-MRS: 51.7; S-MRS: 0
500	1.105×10^6	5.327×10^3	Directly exposed: 33.1; OS: 0.3; P-MRS: 0.5; S-MRS: 6.9
1000	2.211×10^6	10.653×10^3	Directly exposed: 13.2; OS: 1.6; P-MRS: 0.1; S-MRS: 3.1

References

1. Böttger, U.; de Vera, J.-P.; Fritz, J.; Weber, I.; Hübers, H.-W.; Schulze-Makuch, D. Optimizing the detection of carotene in cyanobacteria in a martian regolith analogue with a Raman spectrometer for the ExoMars mission. *Planet. Space Sci.* **2012**, *60*, 356–362.
2. Ehresmann, B.; Zeitlin, C.; Hassler, D.M.; Wimmer-Schweingruber, R.F.; Böhm, E.; Böttcher, S.; Martin, C. Charged particle spectra obtained with the Mars Science Laboratory Radiation Assessment Detector (MSL/RAD) on the surface of Mars. *J. Geophys. Res. Planets* **2014**, *119*, 468–479.

Publisher's Note: MDPI stays neutral with regard to jurisdictional claims in published maps and institutional affiliations.

© 2020 by the authors. Licensee MDPI, Basel, Switzerland. This article is an open access article distributed under the terms and conditions of the Creative Commons Attribution (CC BY) license (http://creativecommons.org/licenses/by/4.0/).
